# Supplementary material for: Learning and memory function in young people with and without perinatal HIV in England
Source: PLoS One. 2022 Sep 15;17(9):e0273645. doi: 10.1371/journal.pone.0273645 (PMC9477265; doi:10.1371/journal.pone.0273645)
Supplement: S1 Table — Mean scores were compared across the three groups (HIV-, PHIV/no C, PHIV/C) using ANOVA tests. Abbreviations: HIV-, HIV negative; PHIV, perinatal HIV; PHIV/C, PHIV with a CDC C diagnosis; PHIV/no C, PHIV without a CDC C diagnosis. (DOCX) [file pone.0273645.s001.docx]

**S1 Table: Cognitive function test, standardised and z-scores by domain, HIV and CDC status**

| Domain/Test | Mean score (standard deviation) [IQR] | | |
| --- | --- | --- | --- |
|  | HIV- (n=68) | PHIV/no C (n=179) | PHIV/C (n=55) |
| Executive function |  |  |  |
| z-score | -1.0 (0.9) [-1.7,-0.4] | -0.9 (0.8) [-1.5,-0.4] | -1.1 (0.8) [-1.6,-0.6] |
| standardised score | 85.6 (12.9) [74.7,93.8] | 86.0 (12.2) [77.9,94.5] | 82.8 (12.1) [75.4,90.4] |
| Flanker inhibitory control and attention |  |  |  |
| test score | 19.9 (0.2) [20.0,20.0] | 19.9 (0.5) [20.0,20.0] | 19.6 (1.1) [20.0,20.0] |
| z-score | -1.1 (0.9) [-1.8,-0.3] | -1.1 (0.8) [-1.6,-0.5] | -1.2 (0.8) [-1.6,-0.7] |
| standardised score | 83.9 (13.9) [73.1,95.0] | 83.9 (12.7) [76.6,92.3] | 81.3 (12.3) [75.6,89.9] |
| Dimensional change card sort |  |  |  |
| test score | 28.9 (1.4) [28.0,30.0] | 28.6 (2.3) [28.0,30.0] | 28.0 (3.4) [28.0,30.0] |
| z-score | -0.8 (1.0) [-1.6,-0.3] | -0.8 (1.0) [-1.6,0.1] | -1.0 (1.0) [-1.9,-0.3] |
| standardised score | 87.3 (14.4) [75.8,94.8] | 88.1 (15.2) [76.1,100.9] | 84.3 (15.6) [72.0,95.7] |
| Speed of information processing |  |  |  |
| test score | 55.9 (14.9) [45.0,67.5] | 51.9 (14.3) [43.0,62.0] | 51.2 (10.7) [45.0,55.0] |
| z-score | -0.6 (1.5) [-1.6,0.4] | -1.0 (1.4) [-1.8,-0.1] | -1.1 (1.1) [-1.7,-0.6] |
| standardised score | 90.5 (21.9) [75.6,106.7] | 84.5 (20.7) [72.7,97.9] | 84.0 (16.2) [74.9,90.9] |
| Verbal learning |  |  |  |
| test score | 23.9 (4.8) [20.0,28.0] | 22.8 (4.6) [20.0,26.0] | 20.9 (5.4) [17.0,25.0] |
| z-score | -1.0 (1.1) [-1.8,-0.3] | -1.3 (1.0) [-2.1,-0.5] | -1.5 (1.1) [-2.4,-0.9] |
| standardised score | 85.0 (17.0) [72.5, 95.8] | 81.2 (15.1) [68.9,92.5] | 77.3 (16.0) [63.6,86.9] |
| Verbal delayed recall |  |  |  |
| test score | 8.1 (2.7) [6.0,10.0] | 7.6 (2.4) [6.0,9.0] | 6.5 (2.8) [5.0,9.0] |
| z-score | -1.1 (1.2) [-1.9,-0.1] | -1.4 (0.9) [-2.0,-0.8] | -1.7 (1.1) [-2.6,-1.1] |
| standardised score | 84.1 (18.1) [71.0,97.8] | 79.5 (14.1) [70.6,87.8] | 74.7 (16.0) [60.7,84.1] |
| Memory |  |  |  |
| z-score | -0.2 (0.9) [-0.8,0.2] | -0.3 (0.8) [-0.8,0.3] | -0.5 (0.9) [-1.0,0.3] |
| standardised score | 97.0 (12.8) [88.4,103.7] | 96.1 (11.9) [87.8,103.8] | 92.9 (13.4) [84.7,104.1] |
| List sorting |  |  |  |
| test score | 17.8 (3.1) [15.0,20.0] | 17.1 (2.8) [15.0,19.0] | 16.5 (3.4) [15.0,19.0] |
| z-score | -0.1 (1.1) [-0.8,1.0] | -0.3 (1.0) [-1.0,0.3] | -0.5 (1.1) [-1.0,0.4] |
| standardised score | 98.6 (15.8) [87.9,114.5] | 95.6 (14.4) [84.3,105.2] | 93.0 (16.4) [84.8,105.4] |
| Picture sequence |  |  |  |
| test score | 18.8 (7.4) [13.0,24.0] | 18.4 (7.5) [13.0,24.0] | 15.9 (7.3) [10.0,21.0] |
| z-score | -0.3 (1.1) [-0.9,0.4] | -0.2 (1.0) [-0.9,0.4] | -0.5 (0.9) [-1.2,0.2] |
| standardised score | 95.5 (15.9) [86.5,105.6] | 96.6 (14.4) [87.1,105.9] | 92.8 (14.2) [81.9,103.1] |
| Verbal application measures |  |  |  |
| z-score | -0.1 (0.9) [-0.5,0.4] | -0.2 (0.8) [-0.9,0.3] | -0.5(0.9) [-1.1,0.3] |
| standardised score | 98.6 (13.1) [92.0,106.5] | 96.3 (12.7) [87.0,105.0] | 92.5 (13.6) [84.0,104.0] |
| WIAT-II |  |  |  |
| test score | 121.5 (9.3) [119.5,127.0] | 119.7 (9.5) [114.0,126.0] | 117.4 (11.0) [110.0,126.0] |
| z-score | 0.5 (1.0) [0.2,1.2] | 0.3 (1.1) [-0.5,1.1] | 0.0 (1.2) [-1.0,0.9] |
| standardised score | 107.7 (15.5) [103.0,118.0] | 104.1 (16.8) [93.0,116.0] | 100.1 (18.6) [85.0,114.0] |
| BPVS III |  |  |  |
| test score | 141.6 (16.5) [134.0,154.5] | 142.5 (13.2) [135.0,151.0] | 137.4 (16.0) [130.0,151.0] |
| z-score | -0.7 (0.9) [-1.4,0.0] | -0.8 (0.8) [-1.4,0.3] | -1.0 (0.8) [-1.7,-0.3] |
| standardised score | 89.5 (13.6) [79.5,99.5] | 88.4 (11.7) [79.0,96.0] | 84.9 (11.9) [75.0,96.0] |
