# Supplementary material for: Learning and memory function in young people with and without perinatal HIV in England
Source: PLoS One. 2022 Sep 15;17(9):e0273645. doi: 10.1371/journal.pone.0273645 (PMC9477265; doi:10.1371/journal.pone.0273645)
Supplement: S2 Table — All a priori variables, as well as those with univariable P-value <0.15 and multivariable P-value <0.05 are presented. Abbreviations: CI, confidence interval, HIV-, HIV negative; PHIV, perinatal HIV; PHIV/C, PHIV with a CDC C diagnosis; PHIV/no C, PHIV without a CDC C diagnosis. (DOCX) [file pone.0273645.s002.docx]

**S2 Table: Predictors of improved verbal learning, verbal delayed recall and executive function scores**

| Variable | Verbal learning | | | Verbal delayed recall | | |
| --- | --- | --- | --- | --- | --- | --- |
|  | Coefficient | 95% CI | P-value | Coefficient | 95% CI | P-value |
| Constant | 22.38 | 18.34, 26.41 |  | 12.0 | 8.19, 15.86 |  |
|  |  |  |  |  |  |  |
| HIV/CDC status (versus HIV-) |  |  | 0.011 |  |  | 0.040 |
| PHIV/no C | -0.76 | -2.10, 0.59 |  | -0.37 | -1.08, 0.34 |  |
| PHIV/C | -2.55 | -4.25, -0.85 |  | -1.18 | -2.10, -0.25 |  |
|  |  |  |  |  |  |  |
| ***Sociodemographics:*** |  |  |  |  |  |  |
| Sex, female (versus male) | 0.59 | -0.53, 1.71 | 0.299 | 0.15 | -0.45, 0.75 | 0.620 |
|  |  |  |  |  |  |  |
| Age at interview2, per year increase | 0.18 | -0.02, 0.37 | 0.085 | 0.08 | -0.03, 0.19 | 0.159 |
|  |  |  |  |  |  |  |
| Black ethnicity (versus non-Black) | -2.59 | -4.11, -1.06 | <0.001 | -1.53 | -2.34, -0.73 | <0.001 |
|  |  |  |  |  |  |  |
| Born outside UK/Ireland (versus born in UK/Ireland) | -0.53 | -1.64, 0.59 | 0.351 | -0.45 | -1.07, 0.17 | 0.156 |
|  |  |  |  |  |  |  |
| ***Lifestyle:*** |  |  |  |  |  |  |
| Waist to hip ratio, per 0.1 increase | - | - | - | -4.76 | -8.63, -0.88 | 0.016 |
|  |  |  |  |  |  |  |

| Variable | Executive function | | | | | |
| --- | --- | --- | --- | --- | --- | --- |
|  | Flanker inhibitory control and attention | | | Dimensional change card sort | | |
|  | Coefficient | 95% CI | P-value | Coefficient | 95% CI | P-value |
| Constant | 7.50 | 6.76, 8.25 |  | 6.83 | 5.99, 7.67 |  |
|  |  |  |  |  |  |  |
| HIV/CDC status (versus HIV-) |  |  | 0.136 |  |  | 0.145 |
| PHIV/no C | 0.01 | -0.24, 0.26 |  | 0.00 | -0.29, 0.30 |  |
| PHIV/C | -0.26 | -0.58, -0.06 |  | -0.29 | -0.65, 0.07 |  |
|  |  |  |  |  |  |  |
| ***Sociodemographics:*** |  |  |  |  |  |  |
| Sex, female (versus male) | -0.26 | -0.47, -0.06 | 0.013 | -0.18 | -0.42, 0.05 | 0.123 |
|  |  |  |  |  |  |  |
| Age at interview 2, per year increase | 0.04 | 0.00, 0.08 | 0.033 | 0.09 | -0.67, -0.04 | <0.001 |
|  |  |  |  |  |  |  |
| Black ethnicity (versus non-Black) | -0.30 | -0.58, -0.01 | 0.040 | -0.35 | -0.67, -0.04 | 0.029 |
|  |  |  |  |  |  |  |
| Born outside UK/Ireland (versus born in UK/Ireland) | 0.05 | -0.16, 0.26 | 0.646 | -0.08 | -0.31, 0.16 | 0.528 |
|  |  |  |  |  |  |  |
| ***Lifestyle:*** |  |  |  |  |  |  |
| Ever smoked (versus never smoked) | 0.22 | 0.01, 0.43 | 0.039 | - | - | - |
|  |  |  |  |  |  |  |
| ***Psychosocial:*** |  |  |  |  |  |  |
| Death of one/both parents (versus both parents alive) | - | - | - | -0.32 | -0.56, -0.09 | 0.007 |
|  |  |  |  |  |  |  |
| ***Environmental:*** |  |  |  |  |  |  |
| Young carer (versus not a young carer) | - | - | - | -0.39 | -0.72, -0.05 | 0.024 |
|  |  |  |  |  |  |  |
