# Supplementary material for: Learning and memory function in young people with and without perinatal HIV in England
Source: PLoS One. 2022 Sep 15;17(9):e0273645. doi: 10.1371/journal.pone.0273645 (PMC9477265; doi:10.1371/journal.pone.0273645)
Supplement: S3 Table — All a priori variables, as well as those with univariable P-value <0.15 and multivariable P-value <0.05 are presented. Abbreviations: CI, confidence interval, HIV-, HIV negative; PHIV, perinatal HIV; PHIV/C, PHIV with a CDC C diagnosis; PHIV/no C, PHIV without a CDC C diagnosis; NNRTI, non-nucleoside reverse transcriptase inhibitor; PI, protease inhibitor; NRTI, nucleoside reverse transcriptase inhibitor. a within 6 months before or after interview. (DOCX) [file pone.0273645.s003.docx]

**S3 Table: Predictors of improved verbal learning, verbal delayed recall and executive function scores for PHIV participants**

| Variable | Verbal learning | | | Verbal delayed recall | | |
| --- | --- | --- | --- | --- | --- | --- |
|  | Coefficient | 95% CI | P-value | Coefficient | 95% CI | P-value |
| Constant | 19.46 | 14.11, 24.81 |  | 7.90 | 5.21, 10.59 |  |
|  |  |  |  |  |  |  |
| HIV/CDC status: PHIV/C (versus PHIV/no C) | -1.83 | -3.33, -0.32 | 0.018 | -0.83 | -1.62, -0.36 | 0.041 |
| ***Sociodemographics:*** |  |  |  |  |  |  |
| Sex, female (versus male) | 1.33 | 0.07, 2.65 | 0.049 | 0.28 | -0.42, 0.97 | 0.431 |
|  |  |  |  |  |  |  |
| Age at interview 2, per year increase | 0.21 | -0.05, 0.46 | 0.111 | 0.01 | -0.12, 0.15 | 0.852 |
|  |  |  |  |  |  |  |
| Black ethnicity (versus non-Black) | -2.57 | -4.53, -0.62 | 0.010 | -1.62 | -2.62, -0.63 | 0.001 |
|  |  |  |  |  |  |  |
| Born outside UK/Ireland (versus born in UK/Ireland) | -0.60 | -1.94, 0.74 | 0.377 | -0.03 | -0.77, 0.72 | 0.946 |
| ***HIV related:*** |  |  |  |  |  |  |
| Viral load <50 copies/ml at interview^a^ | 2.16 | 0.81,3.51 | 0.002 | - | - | - |
|  |  |  |  |  |  |  |
| Class at ART initiation (versus NNRTI based) |  |  |  |  |  | 0.011 |
| Boosted PI based | - | - | - | 1.48 | 0.51, 2.45 |  |
| Unboosted PI based/NRTI only | - | - | - | 0.43 | -0.45, 1.32 |  |
| ***Psychosocial:*** |  |  |  |  |  |  |
| Death of one/both parents death (versus both parents alive) | - | - | - | 0.71 | 0.02, 1.40 | 0.044 |

| Variable | Executive function | | | | | |
| --- | --- | --- | --- | --- | --- | --- |
|  | Flanker inhibitory control and attention | | | Dimensional change card sort | | |
|  | Coefficient | 95% CI | P-value | Coefficient | 95% CI | P-value |
| Constant | 7.37 | 6.50, 8.24 |  | 6.92 | 5.82, 8.02 |  |
|  |  |  |  |  |  |  |
| HIV/CDC status: PHIV/C (versus PHIV/no C) | -0.22 | -0.48, 0.04 | 0.093 | -0.31 | -0.64, 0.02 | 0.068 |
| ***Sociodemographics:*** |  |  |  |  |  |  |
| Sex, female (versus male) | -0.43 | -0.65, -0.20 | <0.001 | -0.24 | -0.52, 0.05 | 0.102 |
|  |  |  |  |  |  |  |
| Age at interview 2, per year increase | 0.06 | 0.02, 0.11 | 0.003 | 0.07 | 0.02, 0.13 | 0.008 |
|  |  |  |  |  |  |  |
| Black ethnicity (versus non-Black) | -0.18 | -0.50, 0.14 | 0.276 | -0.30 | -0.70, 0.11 | 0.157 |
|  |  |  |  |  |  |  |
| Born outside UK/Ireland (versus born in UK/Ireland) | 0.09 | -0.14, 0.32 | 0.447 | -0.17 | -0.45, 0.12 | 0.254 |
| ***HIV related:*** |  |  |  |  |  |  |
| Years viral load <50 copies/ml, per year increase | -0.04 | -0.06, -0.01 | 0.008 | - | - | - |
