# Supplementary material for: Learning and memory function in young people with and without perinatal HIV in England
Source: PLoS One. 2022 Sep 15;17(9):e0273645. doi: 10.1371/journal.pone.0273645 (PMC9477265; doi:10.1371/journal.pone.0273645)
Supplement: Attachment — Submitted filename: Response to Reviewers v1.0.docx [file pone.0273645.s004.docx]

PONE-D-21-16526

**Learning and memory function in young people with and without perinatal HIV in England**

PLOS ONE

We thank the journal and reviewers for their time considering our paper. We are delighted with the opportunity of submitting a revised version of our manuscript for your consideration and, following your request, we have addressed the comments made by your editorial office and the reviewers below.

Academic editor comments:

1. The analyses section needs reconsideration and cleaning up. All data should be provided so that readers can make their own determination and the combining of test scores should be given more thought.  This was seen as a minor revision by Reviewer one and as a major revision by Reviewer 2 but both believed that this aspect of the study could be much improved and that a more sophisticated and nuanced approach to test interpretation was needed.

Response: More detail has been added to the methods and results sections, including the addition of supplementary tables detailing the raw data from the individual tests and results of the regression analyses. We have also added more details on the rationale of combining some of the tests into domains.

2. Both reviewers also questioned whether the introduction adequately positioned the study within the literature and reviewer 2 wanted an indication of what this study adds to your previous reporting of the same data set.

Response: We have added more details to the introduction about our previous study looking at cognitive function at interview 1.

Please see our responses to individual comments below.

Journal Requirements:

1. Please ensure that your manuscript meets PLOS ONE's style requirements, including those for file naming. The PLOS ONE style templates can be found at
   [https://journals.plos.org/plosone/s/file?id=wjVg/PLOSOne_formatting_sample_main_body.pdf](https://eur01.safelinks.protection.outlook.com/?url=https%3A%2F%2Fjournals.plos.org%2Fplosone%2Fs%2Ffile%3Fid%3DwjVg%2FPLOSOne_formatting_sample_main_body.pdf&data=04%7C01%7Ch.castro%40ucl.ac.uk%7C63d8656d2d6f4e205df508d9b8ccc780%7C1faf88fea9984c5b93c9210a11d9a5c2%7C0%7C0%7C637744011919617290%7CUnknown%7CTWFpbGZsb3d8eyJWIjoiMC4wLjAwMDAiLCJQIjoiV2luMzIiLCJBTiI6Ik1haWwiLCJXVCI6Mn0%3D%7C3000&sdata=Hjb79s3oKjwCA0U3Rqj6hVgNEuHnfgFW3gZU0rAua%2Fw%3D&reserved=0) and 
   [https://journals.plos.org/plosone/s/file?id=ba62/PLOSOne_formatting_sample_title_authors_affiliations.pdf](https://eur01.safelinks.protection.outlook.com/?url=https%3A%2F%2Fjournals.plos.org%2Fplosone%2Fs%2Ffile%3Fid%3Dba62%2FPLOSOne_formatting_sample_title_authors_affiliations.pdf&data=04%7C01%7Ch.castro%40ucl.ac.uk%7C63d8656d2d6f4e205df508d9b8ccc780%7C1faf88fea9984c5b93c9210a11d9a5c2%7C0%7C0%7C637744011919627303%7CUnknown%7CTWFpbGZsb3d8eyJWIjoiMC4wLjAwMDAiLCJQIjoiV2luMzIiLCJBTiI6Ik1haWwiLCJXVCI6Mn0%3D%7C3000&sdata=jWE7Se8bLGWYeox77I4oTOZ5Vp2VzQsXnoX7PM8eupU%3D&reserved=0)

Response: This has been done.

2. Please note that in order to use the direct billing option the corresponding author must be affiliated with the chosen institute. Please either amend your manuscript to change the affiliation or corresponding author, or email us at [plosone@plos.org](mailto:plosone@plos.org) with a request to remove this option.

Response: The corresponding author is affiliated to two Institutes from University College London, the Institute of Clinical Trials and Methodology (ICTM) and the Institute for Global Health (IGH). The MRC Clinical Trials at UCL is based at ICTM. It is not clear to us where the confusion is, but would be happy to work with you to get this sorted.

3. One of the noted authors is a group or consortium "Adolescents and Adults Living with Perinatal HIV (AALPHI) Steering Committee". In addition to naming the author group, please list the individual authors and affiliations within this group in the acknowledgments section of your manuscript. Please also indicate clearly a lead author for this group along with a contact email address.

Response: The membership and lead contact for the AALPHI steering committee is already stated in the Acknowledgements section. We have added a note to the title page to indicate that membership of the group is provided in the Acknowledgements.

4. We note that you have included the phrase “data not shown” in your manuscript. Unfortunately, this does not meet our data sharing requirements. PLOS does not permit references to inaccessible data. We require that authors provide all relevant data within the paper, Supporting Information files, or in an acceptable, public repository. Please add a citation to support this phrase or upload the data that corresponds with these findings to a stable repository (such as Figshare or Dryad) and provide and URLs, DOIs, or accession numbers that may be used to access these data. Or, if the data are not a core part of the research being presented in your study, we ask that you remove the phrase that refers to these data.

Response: The data have now been included in a Supporting Information file (S3 Table).

Reviewers' comments:

Reviewer #1:

Major Revisions

1. The manuscript requires better alignment of aims, methods, and results. For example, the aim in the abstract states, “we assess these functions in young people (YP) with perinatal HIV (PHIV) and a comparable group of HIV-negative (HIV-) YP.” However, the methods described three groups. Additionally, the aim is to compare groups on cognitive functioning, but a regression model is run to assess predictors of cognitive functioning. No objective or hypothesis for the regression analysis is reported, nor are the results of the regression reported in the abstract.

Response: We have amended the abstract and introduction to better reflect the three groups compared in the analysis. We have also included the regression analysis in the abstract and added it as an aim in the introduction.

2. Overall, the findings need to be better contextualized within the broader literature in both the introduction and the discussion. For the introduction, what is already known about the effects of perinatal HIV on cognition in young people? What don’t we know about cognitive functioning in this population? Why is this study important? Moreover, the authors cite a previously published paper from their own working group on cognitive performance in the same cohort. These findings should be reported in the introduction. It is unclear how this study differs from their previously published paper.

Response: As the reviewer mentioned, we have explored cognitive function performance in the AALPHI cohort at baseline. In the initial assessment we found no overall difference in neuropsychological testing performance between young people living with HIV, who have never developed any AIDS-defining complication, and HIV-negative young people. However, performance on tests exploring memory and information processing cognitive domains seem to be poorer in PHIV, particularly those who have experienced AIDS-defining complications in the past. Therefore, the round of neuropsychological assessment we are presenting in this manuscript, focused predominately on learning and memory cognitive function domains because of their implications on educational and employment outcomes.

We have amended the introduction to reflect this.

3. For domains with more than one test, z scores were averaged to create a mean z score for each domain. This seems appropriate; however, the rationale for combining certain tests needs to be made explicit. From a neuropsychological perspective, some of the combinations may not be appropriate. For example:

a. What is the rationale for combining list sorting and picture sequence from the NIHTB? The list sorting test is a verbal working memory task and picture sequence is a visual episodic memory task. Conceptually, a working memory task might be better defined as an executive functioning task. See [https://pubmed.ncbi.nlm.nih.gov/24959983/](https://eur01.safelinks.protection.outlook.com/?url=https%3A%2F%2Fpubmed.ncbi.nlm.nih.gov%2F24959983%2F&data=04%7C01%7Ch.castro%40ucl.ac.uk%7C63d8656d2d6f4e205df508d9b8ccc780%7C1faf88fea9984c5b93c9210a11d9a5c2%7C0%7C0%7C637744011919637291%7CUnknown%7CTWFpbGZsb3d8eyJWIjoiMC4wLjAwMDAiLCJQIjoiV2luMzIiLCJBTiI6Ik1haWwiLCJXVCI6Mn0%3D%7C3000&sdata=T5zV6IvkjMY64z2JWgKB4RdYnH7HiRAWdRC9xn9Rf1w%3D&reserved=0).

b. The picture memory task shows good convergent validity with the RAVLT (a very common list learning test), which is similar to the HVLT (see [https://www.ncbi.nlm.nih.gov/pmc/articles/PMC4254833/](https://eur01.safelinks.protection.outlook.com/?url=https%3A%2F%2Fwww.ncbi.nlm.nih.gov%2Fpmc%2Farticles%2FPMC4254833%2F&data=04%7C01%7Ch.castro%40ucl.ac.uk%7C63d8656d2d6f4e205df508d9b8ccc780%7C1faf88fea9984c5b93c9210a11d9a5c2%7C0%7C0%7C637744011919647271%7CUnknown%7CTWFpbGZsb3d8eyJWIjoiMC4wLjAwMDAiLCJQIjoiV2luMzIiLCJBTiI6Ik1haWwiLCJXVCI6Mn0%3D%7C3000&sdata=ErsUkBs5tS%2FsMsUapv%2Buj%2FlLv3UXIa7eglNcrkw%2Bp0I%3D&reserved=0)). What is the rational for calling the HVLT a learning task and separating it from the other memory tasks? Learning is a component of memory. If the HVLT is impaired this is a component of verbal memory.

Response: We do agree that the findings do not clarify the specific neurocognitive processes most affected by the presence of HIV in the developing brain. The choice of tests recognised the complex interaction between attention, memory, information processing and how these affect learning. The selected battery aimed to provide us with an overall baseline of some of the cognitive processes important for learning and wellbeing, but within the limitations imposed by the wider remit of the study. Note This was the first UK study with a comparison group to collect a wide range of data from long term survivors of perinatally acquired HIV infection.

The decision of combining some of the included tests was based more on functions (e.g. immediate or delayed recall) than specific underlying neurocognitive concepts. However, acknowledging the relevance of the points made by the reviewer, we have included separate scores of each of the tests on the S1 table. The table would provide the reader with the opportunity of exploring performance of participants on each test within each study group.

We grouped the tests into domains based on the rationale alluded above, and used Cronbach α (a measure of internal consistency) to check these assumptions. We have clarified this in the methods section.

4. Although the results for the Verbal Learning PHIV C vs PHIV-C are statistically different, the confidence intervals are completely overlapping. Moreover, the z-scores are very similar (1.3 vs 1.5). This translates to a standard score of 78 vs 81, respectively. Is this really clinically meaningful? These scores may result in very similar behavioral presentation. Effect sizes should be reported to demonstrate magnitude of effects.

Response: The results of the ANOVA analysis show that the average z-scores in the three HIV/CDC groups are statistically significantly different which is also found in the regression analysis where HIV/CDC group was a statistically significant predictor of verbal learning scores. In both analyses PHIV/C had the lowest or predicted the lowest scores, followed by PHIV/no C and then the HIV- group. We have amended the wording of our interpretation of the results to suggest a trend can be seen. We have also presented the test scores by HIV/CDC group in S1 Table and the results of the regression analysis in S2 Table.

5. More clarity is warranted with regard to the aim and methods of the regression analysis:
a. What is the goal of the regression analysis? If part of the primary objective of the paper is to determine what factors might predict poorer cognitive functioning in this group, that should be made explicit in the introduction/aims.

Response: This has been added to the introduction.

b. IQ/education are important predictors of cognitive functioning. Were these controlled for in the regression to determine what factors predict poor outcomes? If not, why?

Response: IQ was not collected as part of the AALPHI study. Highest qualification was collected but was poorly completed with lots of missing data and therefore was not included in the analysis.

c. The authors note that the predictors used in the regression can be found in Table 1. Table 1 has over 20 variables. Are all of these included in the regression? I would think the analysis would be underpowered for that many variables. Please clarify and provide justification for the predictors.

Response: The variables with a * asterisk in Table 1 were included as predictors in the overall regression analysis. We have clarified this in the methods section. The variables chosen were reviewed by the AALPHI Steering Committee and included important variables which have been highlighted in other studies.

d. An additional analysis with only PHIV was conducted. What is the rationale for this? Was this decided a priori or is this a sensitivity analysis? This needed clarification.

Response: This analysis was conducted to investigate the effect of HIV-related factors on cognitive performance and was decided a priori. This has been clarified in the introduction and methods section.

6. Raw score means, SDs, and ranges for all cognitive testing should be presented. This is necessary for interpretation of the results.

Response: These data have now been included in a Supporting Information file (S1 Table).

Minor Revisions by Section

Abstract
1. The title and Abstract background make it sound like only learning and memory was assessed. Consider updating both.

Response: We thank the reviewer for their comment. As the main finding is related to these domains, we think the focus of the title is appropriate. The methods section of the abstract confirms the wider assessments conducted.

2. Acronyms are not defined before use. (e.g., CDC C diagnosis, ART)

Response: Thank you for your comment. They are now defined.

3. The original aim is to compare PHIV to HIV-PV, but there is a three-group comparison reported in results.

Response: We have amended the abstract and introduction to better reflect the three groups compared in the analysis.

4. Results of the linear regressions are not mentioned.

Response: We have included the regression analysis in the abstract and added it as an aim in the introduction.

5. The conclusion made in the abstract that results suggest earlier ART leads to better outcome is not support by the results reported in the abstract. There is no mention of ART in the result section.

Response: The reviewer is right in their observation. Our analysis did not show an association with ART exposure or age of ART initiation. However, our analysis clearly demonstrated previous AIDS-defining conditions differentiated the level of cognitive impairment in our study population. Since the main aim of ART is restoring or preventing deterioration of immune function and hence, preventing opportunistic infections or other AIDS-defining conditions; AIDS-defining conditions can be considered as a surrogate marker for delayed ART initiation which may or may not be due to late diagnosis with HIV infection. Participants with AIDS-defining conditions were mainly started on ART when presenting with these complications. Those participants who were started on ART before their immune functions was severely deteriorated showed better performance in all neuropsychological tests and, taking these two points together, we believe the conclusion is well supported. We have clarified our conclusions in the abstract and discussion.

Introduction
6. The connection to education in the introduction is a little unclear. This might be an important implication of the work; however, it is not directly related to the study aim.

Response: We decided to focus on executive function, verbal learning, and memory because these may impact outcomes in education and employability. We have amended the introduction to reflect this.

7. A rationale should be provided for using the HIV-negative control group. I assume this control group accounts for environmental and psychosocial factors related to HIV exposure. This should be made explicit.

Response: This rationale has been added to the methods section, where we think it fits better.

8. In line with the above, there is clearly good reason for comparing groups with and without a CDC C diagnosis separately, but the rationale should be made explicit.

Response: We have amended the introduction to making the inclusion of all three groups clearer.

9. Why are no a prioiri hypotheses reported?

Response: We have clarified the aims of the analysis in the introduction.

Method
10. The first sentence needs editing. It reads like the study was evaluating the impact of HIV infection and ART on HIV- young people as it is currently written.

Response: This has been amended.

11. Please report any exclusion criteria for the larger study.

Response: This has been added.

12. A lot of space is used to discuss the consent/assent process. This is good/ethical practice, but unnecessarily detailed for a manuscript.

Response: Some of the detail has been removed from this section.

13. Cognitive testing described to have occurred during a face-to-face interview. It may be better described as an interview with cognitive testing.

Response: This has been reworded.

14. Please define the CDC Clinical classification class C past history of an AIDS defining illness.

Response: The CDC C classification includes over 20 conditions. We have referenced the paper listing the conditions (apologies an incorrect reference was used in the original manuscript and has now been updated). If the editor would like all the conditions listed in the manuscript, then we can add them.

Statistical analysis
15. Specify what comparisons are being made using chi square tests/Wilcoxon/KW.

Response: This has been clarified.

Results
16. Were there any significant differences between your groups on important demographic or clinical factors such as education, SES, psychiatric diagnoses? I believe these comparisons are made in Table 1, but the authors should include a statement in the results and then point readers to the table.

Response: A sentence describing the significant differences has been added to the results section and Table 1 referenced.

17. Information on variable distribution/assumptions underlying statistical analyses should be reported.

Response: The normality of the data was assessed using visual plots and Sharpio-Wilk tests. Fisher’s exact tests for proportions were used if less than 80% of the cells had an expected value of five or greater. This information has been added to the Methods section.

Discussion
18. It would be interesting to discuss in more detail why your control group also performed lower than the normative sample on several tests to contextualize the results. For example, what common factors between all three groups could have impacted cognitive performance on these measures and is this consistent with the literature?

Response: Young people recruited as part of the comparison group were mainly siblings of PHIV or had a parent living with HIV and therefore, have been affected by HIV since their early childhood, making them comparable to our PHIV participants as they were likely to be exposed to all unmeasured factors that can affect development of cognitive functions. For instance, the majority of youngsters in both groups will have had a mother who was HIV positive and parental ill health, both physical and mental, and death and loss is much higher than in general population. Inconsistency or changes in care and the emotional and economic consequences of living with chronic parental ill health as well as the anxiety and confusion and uncertainty associated with managing a secret and stigmatising family diagnosis and add to the obstacles faced by many of these youngsters. These early chronic factors affect later learning and psychological outcomes and may explain the finding that scores on many tests fell below the published test norms in all study groups.

We have added more details about the HIV- comparison group to the methods section.

19. Based on what is reported in the discussion, the HIV+ cohort in this study had impairments in cognitive domains that differed from previously published worked. This should be discussed.

Response: Our study reported performance on all cognitive function domains below the normative mean of each test, which is consistent with several previous studies. In the discussion, we speculated unmeasured factors, such as socioeconomic variables, might affect testing performance (we have added a recent study Mbewe et al JAIDS 2022) and stated the importance of including an appropriate comparison group (i.e. HIV exposed but uninfected). Our results are also consistent with previous studies showing poorer performance in PHIV with previous AIDS-defining conditions when compared to those with less severe HIV disease and exposed but uninfected young people.

20. There is a note to add “blackmore reference” in the discussion.

Response: Thanks for pointing this out, it has been removed. The reference has already been included.

21. It is unclear why the second limitation is a limitation of the study. Was identifying differential effect of antiretroviral drugs on cognition a goal? Is there a reason to believe that different treatments affect cognition in different ways? If so, this should be made more explicit.

Response: Virological escape and compartmentalisation have been reported in PHIV. In patients treated with ART combinations with poorer penetration to the CNS or in those with other conditions affecting blood-brain barrier integrity, viral particles and consequent inflammatory mediators can be observed in CSF, even in patients with undetectable HIV-RNA in peripheral blood. Detectable HIV-RNA in CSF has been associated with a number of neurological negative outcomes, including impaired cognition. In addition, some anti-retroviral drugs may cause neuropsychiatric adverse effects some of which may interfere with neuropsychological testing. Therefore, not being able to explore the impact of ART exposure on cognition we believe is a limitation of the study.

22. A limitation of this study is only using active recall to test memory (i.e., no recognition memory test from HVLT), therefore it is unclear if deficits in delayed memory extend to recognition. If this cohort has impaired learning and recall, but not encoding (i.e., recognition memory is average), this would suggest a frontal executive, as opposed to amnestic mesial temporal profile.

Response: We agree with the reviewer, not including the verbal recognition component of the revised HVLT limits our ability of getting a full interpretation of the results if the aim of the analysis was understanding the neurological pathway leading to the observed performance level. However, the testing battery also included the NIH Toolbox Picture Sequence Test to explore episodic memory which measures episodic memory. Episodic memory could inform on medial temporal lobe function.

The neuropsychological test battery used for this analysis was one of several components included in the main study visit, which was decided in advance should not last more than two hours to make it more likely for each participant to complete the full assessment. That was the main reason for restricting the number of neuropsychological tests. In any case, the aim of the analysis conducted was to compare performance on specific cognitive function domains between PHIV and appropriately selected HIV negative young people.

23. The authors conclude that their findings support that earlier ART protects against cognitive development. Was age of ART included in the regression model? Did it predict outcomes? It seems you have the data to test this assumption, but it is not clearly reported anywhere in the manuscript.

Response: We agree with the reviewer (also see earlier response to minor revisions, comment 5), the tone of the conclusion seems to suggest ART initiation at an earlier age, may protect cognitive function. However, the intention was to state ART initiation at an earlier stage of the HIV disease (i.e. before it reaches severe immunosuppression and opportunistic diseases occur) would reduce the risk of cognitive impairment. The text of the manuscript was amended to reflect the basis for the conclusion. Age of ART initiation was included as a predictor in the PHIV only model, but it was not associated with cognitive performance. The methods section has been amended to clarify which variables were considered as predictors in the models.

Tables
24. Table 1 is very large. I would recommend splitting up into more than one table.

Response: The table has been split into Table 1 (Characteristics of all participants) and Table 2 (HIV-related factors for PHIV group).

25. Should present results of the linear regressions in a table for transparency.

Response: The results of the linear regressions have been added as Supplementary Information (S2 and S3 Tables).

Reviewer #2: Abstract

1. Background paragraph of abstract emphasizes relevance of learning and memory for successful educational and career progression, but this was not examined in this study. Authors should consider a more relevant background justification that would more directly apply to this work and supporting analyses.

Response: See response to reviewer 1, minor revisions, comment 6.

2. “CDC C” diagnosis term in abstract would not be known to a general scientific audience. Consider briefly defining.

Response: The definition of CDC C has been added to the abstract.

Methods
1. The section on the consent process can be shortened/streamlined.

Response: Some of the detail has been removed from this section.

2. It would be helpful for the authors to briefly explain why it would be relevant to compare PHIV/no C and PHIV/C. This is done within the discussion, but it would be beneficial for the reader to understand the reasoning for this comparison from the outset (perhaps within the introduction?)

Response: Our group and others have reported advanced HIV disease, defined as CDC stage C, to be an important risk factor for poorer cognitive function. In the same study population during their first neuropsychological assessment, participants in the PHIV/C performed worse than PHIV/no C and HIV- young people in some specific function domains, including executive function, speed of information processing and fine motor skills. Of note, at the initial assessment, we observed no difference between PHIV/no C and HIV- young people in any cognitive domain testing performance (Judd et al Clin Infect Dis 2016). We do agree with the reviewer, the rationale for comparing participants with different CDC status could be better explained on the text and we have amended the introduction accordingly.

3. Executive functioning and memory performance can be directly impacted by attention, and attention difficulties are documented in HIV. Was there a reason attention measures were not included in this battery?

Response: We do agree with the reviewer on the impact attention impairment would have on executive functioning and memory. Due to time constrains we were unable to include a more comprehensive neuropsychological testing battery and therefore, we decided to include tests that could explore additional cognitive function domains. For instance, as part of the tests used to explore executive functioning in our participants, we included the NIH Toolbox Flanker Inhibitory Control and Attention test that directly explores attention. So, if one wanted to explore the impact of attention impairment on the overall assessment of executive functioning, one could look into the Flanker Inhibitory Control test score. However, the aim of the analysis conducted was to compare performance on specific cognitive function domains, including executive functioning, between PHIV and appropriately selected HIV negative young people.

Results
1. While it is logical that multiple test scores within a domain would be averaged to present results in a cohesive manner, the authors should make explicit that the scores were sufficiently statistically similar for this to be appropriate. Please include a table summarizing mean performance per group for all individual tests using a common metric (e.g., z scores). Also explain the criteria for averaging across tests within a domain; for example, did the authors ensure that mean test scores within a domain were not statistically different from each other? I imagine that there may have been some variability in performance for within-domain tests. Is it possible that potential effects were attenuated because of variable performance within domains?

Response: Mean test scores and z-scores for each test have now been included in a Supporting Information file (S1 Table). As stated before, we grouped the tests into domains based on theoretical assumptions and used Cronbach α (a measure of internal consistency) to check these assumptions. We have clarified this in the methods section.

Discussion
1. There were some lower cognitive scores amongst participants of Black race found in adjusted multivariable models. It is important that the authors address potential explanations for this within the discussion.

Response: This follows a trend reported in our previous paper and we outline potential reasons for this trend in our previous paper. We have added a summary comment to the discussion of this paper.

General comment for consideration:

1. The term “Black” generally refers to a race/racial category, as you can have participants who are “Black” but report different ethnic backgrounds. The authors can consider these recently published suggestions for reporting of race or ethnicity: [https://jamanetwork.com/journals/jama/fullarticle/2776936](https://eur01.safelinks.protection.outlook.com/?url=https%3A%2F%2Fjamanetwork.com%2Fjournals%2Fjama%2Ffullarticle%2F2776936&data=04%7C01%7Ch.castro%40ucl.ac.uk%7C63d8656d2d6f4e205df508d9b8ccc780%7C1faf88fea9984c5b93c9210a11d9a5c2%7C0%7C0%7C637744011919647271%7CUnknown%7CTWFpbGZsb3d8eyJWIjoiMC4wLjAwMDAiLCJQIjoiV2luMzIiLCJBTiI6Ik1haWwiLCJXVCI6Mn0%3D%7C3000&sdata=Rj7YQ36fxk%2BIE516r9sb977kdlbPqTO5h0e8tFkcdww%3D&reserved=0)

Response: We have added to the methods section that ethnicity was self-reported, and clarified the categories used and which categories were combined for the purpose of the analysis. We have also capitalised the names of ethnicities. We have not reported detailed ethnicity breakdowns due to small numbers in some categories.
