# Supplementary material for: Learning and memory function in young people with and without perinatal HIV in England
Source: PLoS One. 2022 Sep 15;17(9):e0273645. doi: 10.1371/journal.pone.0273645 (PMC9477265; doi:10.1371/journal.pone.0273645)
Supplement: Attachment — Submitted filename: Justification for response to reviewers (v1.0; 18MAY2022).docx [file pone.0273645.s005.docx]

**Appeal request form - Response to Reviewer #1: PONE-D-21-16526R1**

We provide a point-by-point response to the reviewer here. The reviewer’s concerns are in **bold text** and our response in *italics*.

As a way of background, in December 2021 we received numerous comments from two reviewers as well as a summary from the Academic Editor, in which the Editor noted that

**“The reviewers and I agree that this work is of importance and that it should be published but both reviewers have highlighted areas where the paper can be strengthened to have a greater impact.”**

This implies that the manuscript was already publishable but that improvements could lead to a stronger paper. We responded robustly to all of the comments, and made significant changes to the manuscript, although in hindsight could have improved the introduction further.

**The authors appropriately addressed many of the minor concerns and clarifications recommended by the reviewers; however, there are a few major concerns that were not addressed in this revision.**

**1. A more thorough review of the background literature would be helpful.**

*In our last revision, we added more details to the introduction about our previous study looking at cognitive function at interview 1.*

*We have now added references and text to contextualise our study within recent literature. See tracked changes attached.*

**2. It is still not clear how this study adds to the previously published study. It sounds like the previous study included the same three groups and a variety of cognitive measures (presumably EF, learning, and memory measures). Is this a replication?**

*We actually wanted to measure for a second time performance in some of the cognitive functions we explored in interview 1, which were most affected in interview 1, but we decided not to look at change over time on any of these domains because we were using different tests. We decided to use different tests to increase comparability of our cohort to a similar study in the USA.*

*In our previous response to reviewer comments we said:*

*“As the reviewer mentioned, we have explored cognitive function performance in the AALPHI cohort at baseline. In the initial assessment we found no overall difference in neuropsychological testing performance between young people living with HIV, who have never developed any AIDS-defining complication, and HIV-negative young people. However, performance on tests exploring memory and information processing cognitive domains seem to be poorer in PHIV, particularly those who have experienced AIDS-defining complications in the past. Therefore, the round of neuropsychological assessment we are presenting in this manuscript, focused predominately on learning and memory cognitive function domains because of their implications on educational and employment outcomes. We have amended the introduction to reflect this.”*

*As stated above, we have already reported some differences in cognitive testing performance between participants with previous AIDS-defining conditions compared to those living with HIV but that have never progressed to CDC stage C conditions. The aim of this second neuropsychological assessment was to further explore cognitive domains considered particularly relevant to school progression and transition to adulthood. The assessment conducted is NOT a replication of our previous analysis.*

*We therefore have made no further changes.*

**3. I still have concerns about the combination of neuropsychological tests scores. The authors have grouped the tests “based on theoretical assumptions”, but there is empirical evidence to suggest different groupings would make more sense based on the underlying neurocognitive functions assessed by these measures. The rationale for the groups needs to be strengthened.**

*We believe that the reviewer has strong feelings against grouping tests in a way different to what they think is appropriate. We do not think it should be up to the reviewer to decide what rationale for combining tests is valid or not, but despite our explanations on the matter, they still seem unhappy.*

*To clarify, we used more than one test to explore three of the cognitive function domains evaluated. When exploring Executive Functioning, we used two NIH ToolBox tests that are designed and validated for that use (i.e. flanker inhibitory control and attention test and the dimensional change card sort test. The latest one, specifically designed and validated to explore cognitive flexibility).*

*When exploring language-related functions, we used the Weschler Individual Achievement Test® and the* British Picture Vocabulary Scale, both commonly used and validated tests for this purpose.

Finally, because focusing on memory and learning skills was our main aim, we did conduct a number of different tests exploring that function domain. However, precisely because we wanted to investigate performance on each one of the components or interrelated specific tasks, we reported separately scores on verbal learning, verbal recall (HVLT-R) and episodic memory (NIH ToolBox: list sorting and picture sequence test). We also reported performance on attention and information processing speed separately.

Therefore, we seriously struggle to understand the objections the reviewer may have on our approach to report performance by cognitive function domain. We have however amended the methods section to reflect the rationale for grouping neuropsychological tests (lines 125 and 126 of the amended draft).

*We previously responded to this comment with the following:*

*“We do agree that the findings do not clarify the specific neurocognitive processes most affected by the presence of HIV in the developing brain. The choice of tests recognised the complex interaction between attention, memory, information processing and how these affect learning. The selected battery aimed to provide us with an overall baseline of some of the cognitive processes important for learning and wellbeing, but within the limitations imposed by the wider remit of the study. Note This was the first UK study with a comparison group to collect a wide range of data from long term survivors of perinatally acquired HIV infection. The decision of combining some of the included tests was based more on functions (e.g. immediate or delayed recall) than specific underlying neurocognitive concepts. However, acknowledging the relevance of the points made by the reviewer, we have included separate scores of each of the tests on the S1 table. The table would provide the reader with the opportunity of exploring performance of participants on each test within each study group. We grouped the tests into domains based on the rationale alluded above, and used Cronbach α (a measure of internal consistency) to check these assumptions. We have clarified this in the methods section.”*

**4. The authors did not address the concern regarding the difference between statistical and clinical significance. Adding effect sizes would help readers understand the magnitude of effect for the statistically significant group difference.**

*We did not see any concerns regarding the difference between statistical and clinical significance in the previous set of reviewer comments. We responded to the request to add effect sizes in our previous response. We added further information on significant differences, and we added a supplementary table showing separate scores of each test. The discussion of the results does not include any speculation on the clinical impact of the differences we observed between the study groups on specific neuropsychological testing scores. Rather, we provided the reader with the scores by test and cognitive functional domain. Exploring the clinical impact of the differences observed was beyond the scope of this analysis and therefore, we have not made any further changes here.*
