# Supplementary material for: Learning and memory function in young people with and without perinatal HIV in England
Source: PLoS One. 2022 Sep 15;17(9):e0273645. doi: 10.1371/journal.pone.0273645 (PMC9477265; doi:10.1371/journal.pone.0273645)
Supplement: Attachment — Submitted filename: PlosOneReview.docx [file pone.0273645.s006.docx]

PONE-D-21-16526R2

1. The introduction and background review has been improved.

2. I am not doubting that the study adds to the literature. My confusion on what this study adds to their previous work comes from the lack of specificity in Line 70-77 (bolded).

“We have previously reported similar performance in neuropsychological testing in young people living with HIV, without a prior history of AIDS-defining complications, and a comparable group of HIV negative (HIV-) young people (YP) within the Adolescents and Adults Living with Perinatal HIV (AALPHI) cohort study (7). Of note, PHIV with a past history of AIDS74

defining conditions **showed poorer performance in all cognitive function domains explored.** In this paper we report on **further cognitive testing performance** of young people with PHIV

(with or without a previous AIDS-defining illness) and a comparable 76 group of HIV- young people.”

The difference between their first and second study is made clearer in their response to reviewers, but the rationale has still not been added to the introduction for readers. If I am interpreting correctly, the authors were (1) further clarifying the differences in memory and information processing found in previous work and (2) used tests more widely used in the literature to compare to other findings. If I am interpreting this correctly, the authors could add this rationale into the manuscript to increase clarity for readers.

3. There is no one valid way to combine neuropsychological test scores. I provided empirical literature regarding the underlying neurocognitive functions that those tests measure. I did not state the authors must group tests as I suggested, only that it should be considered. For example, “verbal delayed recall” and “memory” tests are reported separately, but verbal delayed recall is memory. The authors show that the mean and Z-scores for the combined tests are comparable, which is helpful. Perhaps changing the name of the categories to better reflect the function they are attempting to assess would help.

4. Including the raw cognitive test data was very helpful. However, adding standardized effect sizes along with statistical significance testing demonstrates the magnitude of group difference and is common practice.

https://www.ncbi.nlm.nih.gov/pmc/articles/PMC3444174/#:~:text=Effect%20size%20helps%20readers%20understand,the%20Abstract%20and%20Results%20sections.
